# Factors associated with orthostatic hypotension presence and severity in multiple system atrophy: a cross-sectional study

**DOI:** 10.3389/fneur.2026.1950923

**Published:** 2026-09-17

**Authors:** Kaige Zhang, Xuan Xu, Yao Wang, Han Zhang, Liye Wang, Yunxian Wang, Meiqi Wei, Nannan Li, Zhigang Chen

**Affiliations:** 1 The Second Clinical College, Beijing University of Chinese Medicine, Beijing, China; 2 Department of Neurology 1, The Dongfang Hospital, Beijing University of Chinese Medicine, Beijing, China; 3 Department of Acupuncture, Shaanxi Provincial People’s Hospital, Xi’an, China

**Keywords:** autonomic dysfunction, cross-sectional study, multiple system atrophy, non-motor symptoms, orthostatic hypotension

## Abstract

**Background:**

The factors governing the presence and severity of orthostatic hypotension (OH) in multiple system atrophy (MSA) may differ. We investigated factors independently associated with OH and severe OH in MSA, and assessed the consistency of these associations across subtypes.

**Methods:**

We examined the clinical data of 242 MSA patients who had blood pressure evaluations to assess OH. We analyzed the clinical data of MSA patients with and without OH, as well as those with mild and severe OH, focusing on clinical characteristics and brain MRI features to identify factors associated with OH presence and severe OH.

**Results:**

OH was present in 136 patients (56.2%). MSA-C subtype (OR = 2.42, 95% CI = 1.33–4.43, *p* = 0.004), probable MSA (OR = 2.68, 95% CI = 1.49–4.80, *p* = 0.001), and higher UMSARS-I scores (OR = 1.10, 95% CI = 1.03–1.17, *p* = 0.005) were independently associated with the presence of OH. Among OH-positive patients, male (OR = 2.57, 95% CI = 1.21–5.44, *p* = 0.014) and higher NMSS scores (OR = 1.06, 95% CI = 1.02–1.11, *p* = 0.009) were independently associated with severe OH, whereas MSA subtype was not. Subgroup analyses showed directionally consistent associations for male sex and NMSS across MSA-C and MSA-P subgroups. The association between NMSS and severe OH was statistically significant in the MSA-C subgroup (OR = 1.09, 95% CI = 1.03–1.15, *p* = 0.004), whereas the remaining subgroup estimates did not reach statistical significance. No neuroimaging marker was associated with OH.

**Conclusion:**

Factors associated with OH presence differed from those associated with severe OH in MSA. MSA-C subtype, diagnostic certainty, and UMSARS-I score were associated with OH presence, whereas male sex and NMSS burden were associated with severe OH.

## 1 Introduction

Multiple system atrophy (MSA) is a rare and fatal neurodegenerative disease characterized by the accumulation of α-synuclein-containing oligodendroglial cytoplasmic inclusions (1). Based on clinical symptoms, this neurodegenerative disorder can be classified into a parkinsonian subtype (MSA-P) and a cerebellar subtype (MSA-C) (2). MSA advances rapidly, with a median duration from onset to wheelchair reliance and bed confinement of 3.5 years and 5 years, respectively (3), and a median survival period of under 9 years (4), significantly impacting patients’ longevity and quality of life. Currently, there is no effective treatment.

As a prominent characteristic of autonomic dysfunction in MSA, orthostatic hypotension (OH) is defined as a reduction in blood pressure (BP) of at least 20/10 mmHg (systolic/diastolic) upon changing from a supine to an upright position (5). The prevalence of OH in MSA ranges from 54 to 80% (6, 7), with clinical presentations that are intricate and varied, including dizziness or vertigo, cognitive deterioration, falls, and syncope (8), rendering it the most debilitating complication in MSA (9). Research indicates that the level of OH is markedly correlated with the severity of the condition (10). Earlier onset of OH correlates with a reduced survival duration, and the mortality risk is markedly elevated in patients experiencing severe OH (11, 12). Asymptomatic OH can elevate the likelihood of negative outcomes (8). Consequently, early identification is essential for clinical intervention. We intend to examine the clinical characteristics of MSA patients and analyze variables linked to OH positivity for early prevention and treatment.

## 2 Materials and methods

### 2.1 Patients

Patients who met the second consensus criteria for possible or probable MSA (13) were recruited from Dongfang Hospital, Beijing University of Chinese Medicine, between 2015 and 2025. The exclusion criteria included: (1) severe cardiovascular or cerebrovascular diseases with significant organ dysfunction, (2) active malignancy, and (3) serious psychiatric problems or cognitive impairment that would hinder study participation. The Ethics Committee of Dongfang Hospital (Approval No. JDF-IRB-2025038903) approved this study, and all patients provided informed permission.

### 2.2 Clinical assessments

We collected comprehensive clinical data, including demographic characteristics (sex, age at onset, and disease duration), medical history, history of toxic exposure, orthostatic symptoms, rapid eye movement sleep behavior disorder (RBD), urge incontinence, and constipation.

Blood pressure (BP) was evaluated using a standardized method. BP was assessed while in the supine position after 10 min of resting in a supine posture using a electronic sphygmomanometer. Patients were instructed to rise, and BP was assessed at 1 min and 3 min after standing. The lowest systolic and diastolic blood pressure recorded within the 3-min standing period were used to determine the presence of OH. Orthostatic hypotension (OH) was defined as a decrease in systolic blood pressure (SBP) of ≥20 mmHg and/or a decrease in diastolic blood pressure (DBP) of ≥10 mmHg within 3 min of standing from the supine position. Mild OH was defined as a decrease in SBP of 20–29 mmHg and/or a decrease in DBP of 10–14 mmHg, whereas severe OH was defined as a decrease in SBP of ≥30 mmHg and/or a decrease in DBP of ≥15 mmHg within 3 min of standing (13).

RBD was diagnosed by a structured clinical interview conducted by a senior neurologist. According to the second consensus statement (13), age at onset was defined as the onset of either motor symptoms (parkinsonism or cerebellar ataxia) or specific autonomic symptoms, including unexplained urinary urgency, urinary frequency, incomplete bladder emptying, or OH. For descriptive and subgroup analyses, patients were categorized according to disease duration as <3 years, 3 to <5 years, and ≥5 years. This categorization was based on the reported median intervals from symptom onset to wheelchair dependence (3.5 years) and bed confinement (5 years) in the natural history of MSA (3).

The Unified MSA Rating Scale (UMSARS) was employed to evaluate disease severity and motor symptoms, with UMSARS-I (12 items, each scored 0–4; total score 0–48) assessing activities of daily living and UMSARS-II (14 items, each scored 0–4; total score 0–56) measuring motor function. The total UMSARS score was the sum of part I and part II, and higher scores indicated greater impairment. UMSARS-IV was used to measure global impairment on a scale from 1 to 5 (14). The Non-Motor Symptoms Scale (NMSS) was used to evaluate non-motor symptoms, which comprised 30 items across nine domains with a total score of 0–360. The Montreal Cognitive Assessment (MoCA) was used to assess cognitive function with a total score of 30.

### 2.3 Image analysis

All patients underwent standardized brain MRI using a 3.0 T superconducting scanner (GE Healthcare) with predefined T2-weighted and T2-FLAIR sequences. HCBS was classified from 0 to V based on Horimoto’s criteria (15): Grade 0 (normal), Grade I (emerging vertical hyperintensity), Grade II (distinct vertical line), Grade III (additional horizontal hyperintensity), Grade IV (clear cruciform appearance), and Grade V (ventral pontine hyperintensity or atrophy).

The severity of white matter hyperintensities (WMH) was evaluated using the semi-quantitative Fazekas visual rating scale (16, 17), which assessed both periventricular white matter hyperintensities (PWMH) and deep white matter hyperintensities (DWMH). PWMH is scored from 0 to 3 (0 = absent, 1 = caps or pencil-thin lining, 2 = smooth halo, 3 = irregular periventricular extension), while DWMH is also scored from 0 to 3 (0 = absent, 1 = punctate foci, 2 = beginning confluence, 3 = large confluent areas). The cumulative score is classified as mild (0–2), moderate (3–4), or severe (5–6). All MRI images were separately assessed by a senior neuroradiologist who was unaware of the clinical features.

### 2.4 Statistical analysis

Statistical analyses were performed using SPSS 22.0 for Windows (SPSS Inc., Chicago, IL, USA). Continuous variables were expressed as mean ± standard deviation (SD), and categorical variables as counts (percentages). For between-group comparisons, quantitative data were analyzed using independent samples *t*-test or Mann–Whitney *U* test, while qualitative data were analyzed using chi-square test or Fisher’s exact test. Variables with statistical significance (*p* < 0.05) in univariate analysis, as well as those considered clinically relevant (e.g., age, disease duration), were entered into the binary logistic regression model to identify independent factors associated with OH positivity in MSA. A *p* value < 0.05 was considered statistically significant.

## 3 Results

### 3.1 Study population

A total of 242 patients (53.72% male and 46.28% female) were included in this study, of which 61.57% were diagnosed with MSA-C. The UMSARS-IV disability evaluation showed that 62.81% of patients were in stages 1–2, and 86.36% had a disease duration of less than 5 years, with a mean duration of 2.67 years. The demographic and clinical features of the patients are shown in Table 1.

**Table 1:** Demographic and clinical characteristics of the total sample and comparison between patients with and without OH.

Variables	MSA with OH (*n* = 136)	MSA without OH (*n* = 106)	*p*
Gender			0.199
Male	78 (57.4%)	52 (49.1%)	
Female	58 (42.6%)	54 (50.9%)	
Age of onset (year)	56.15 ± 7.21	56.72 ± 7.47	0.549
Disease duration	2.80 ± 1.83	2.50 ± 1.53	0.278
Past history			
Hypertension [*n* (%)]	25 (18.4%)	23 (21.7%)	0.521
Diabetes [*n* (%)]	11 (8.1%)	14 (13.2%)	0.194
Coronary heart disease [*n* (%)]	5 (3.7%)	8 (7.5%)	0.185
Hyperlipemia [*n* (%)]	9 (6.6%)	5 (4.7%)	0.530
Smoking [*n* (%)]	36 (26.5%)	21 (19.8%)	0.226
Drinking [*n* (%)]	41 (30.1%)	35 (33%)	0.633
Toxic exposure [*n* (%)]	11 (8.1%)	5 (4.7%)	0.295
MSA subtype			**0.014**
MSA-C [*n* (%)]	93 (68.4%)	56 (52.8%)	
MSA-P [*n* (%)]	43 (31.6%)	50 (47.2%)	
Possible/probable (*n*)	50/86	70/36	**0.000**
Orthostatic symptoms [*n* (%)]	82 (60.3%)	23 (21.7%)	**0.000**
RBD	102 (75.0%)	80 (75.5%)	0.933
Urge incontinence	83 (61.0%)	50 (47.2%)	**0.032**
Constipation [*n* (%)]	95 (69.9%)	66 (62.3%)	0.215
UMSARS total score	40.20 ± 14.43	37.20 ± 11.14	0.216
UMSARS-0049	21.78 ± 6.76	18.88 ± 5.31	**0.003**
UMSARS-II	18.49 ± 8.38	18.32 ± 6.77	0.822
UMSARS-IV	2.44 ± 1.22	2.25 ± 1.08	0.311
NMSS	35.44 ± 11.79	31.61 ± 12.21	**0.008**
MOCA	23.06 ± 4.51	23.25 ± 4.09	0.941
HCBS [*n* (%)]	82 (60.3%)	66 (62.3%)	0.755
Moderate-to-severe WMH [*n* (%)]	13 (9.6%)	8 (7.5%)	0.581

HCBS, hot cross bun sign; MoCA, Montreal cognitive assessment; MSA, multiple system atrophy; NMSS, non-motor symptoms scale; OH, orthostatic hypotension; RBD, REM sleep behavior disorder; UMSARS, unified MSA rating scale; WMH, white matter hyperintensity. Values are *n* (%) or mean ± SD. *p* values with statistical significance are shown in bold.

### 3.2 Prevalence of OH

Of the 242 patients, 136 (56.20%) had OH. Compared to MSA-P patients, MSA-C patients had a greater prevalence of OH (62.42% vs. 46.24%, *p* = 0.014). The frequency of OH differed statistically between probable and possible MSA patients (70.49% vs. 41.67%, *p* < 0.001). Based on disease duration, all patients were divided into three groups: disease duration <3 years (*n* = 139), disease duration ≤5 years (*n* = 70), and disease duration ≥5 years (*n* = 33). OH prevalence did not differ significantly between patients with disease duration <3 years (54.68%) and ≥5 years (63.64%, *p* = 0.351). The incidence of OH was significantly higher in patients with urge incontinence than in those without (62.41% vs. 48.62%, *p* = 0.032). There was no significant difference in OH prevalence between males and females (60.00% vs. 51.79%, *p* = 0.199).

### 3.3 Factors associated with the presence of OH

Univariate analyses are presented in Table 1. Compared with patients without OH, patients with OH differed significantly in MSA subtype, diagnostic certainty, presence of orthostatic symptoms, urge incontinence, UMSARS-I score, and NMSS score. No significant differences were observed between the two groups regarding gender, age of onset, disease duration, past history, RBD, constipation, UMSARS score, UMSARS-II score, UMSARS-IV score, MoCA score, HCBS or moderate to severe WMH.

The multivariable binary logistic regression model (Table 2) included sex, age at onset, disease duration, MSA subtype, diagnostic certainty, urge incontinence (present vs. absent), UMSARS-I score, and NMSS score. MSA-C subtype (OR = 2.42, 95% CI = 1.33–4.43), probable MSA (OR = 2.68, 95% CI = 1.49–4.80), and higher UMSARS-I scores (OR = 1.10, 95% CI = 1.03–1.17) were independently associated with the presence of OH.

**Table 2:** Factors independently associated with the presence of OH in multivariable binary logistic regression analysis.

Variables	OR	95% CI	*p*
Female	0.69	0.39–1.22	0.203
Age of onset	0.97	0.94–1.01	0.188
Disease duration	0.96	0.81–1.41	0.643
MSA-C	2.42	1.33–4.43	**0.004**
Probable MSA	2.68	1.49–4.80	**0.001**
Urge incontinence	1.47	0.81–2.66	0.200
UMSARS-I	1.10	1.03–1.17	**0.005**
NMSS	1.00	0.97–1.03	0.801

MSA, multiple system atrophy; NMSS, non-motor symptoms scale; UMSARS, unified MSA rating scale. *p* values with statistical significance are shown in bold.

In a sensitivity analysis excluding diagnostic certainty from the multivariable model, MSA-C subtype (OR = 2.63, 95% CI = 1.46–4.72) and UMSARS-I score (OR = 1.11, 95% CI = 1.04–1.78) remained independently associated with the presence of OH.

### 3.4 Clinical characteristics and factors associated with severe OH among patients with OH

Table 3 showed a comparison of clinical features of OH-positive patients with mild versus severe OH. There were statistically significant differences between the mild and severe OH groups on gender, orthostatic symptoms, and NMSS score.

**Table 3:** Comparison of clinical characteristics between patients with mild OH and severe OH.

Variables	Mild OH (*n* = 54)	Severe OH (*n* = 82)	*p*
Gender			**0.034**
Male	25	53	
Female	29	29	
Age of onset (year)	56.28 ± 7.72	56.06 ± 6.91	0.865
Disease duration	2.56 ± 1.82	2.95 ± 1.84	0.155
Past history			
Hypertension [*n* (%)]	11 (20.37%)	14 (17.07%)	0.627
Diabetes [*n* (%)]	4 (7.41%)	7 (8.54%)	1.000
Coronary heart disease [*n* (%)]	4 (7.41%)	1 (1.22%)	0.158
Hyperlipemia [*n* (%)]	6 (11.11%)	3 (3.66%)	0.174
Smoking [*n* (%)]	14 (25.93%)	22 (26.83%)	0.907
Drinking [*n* (%)]	17 (31.48%)	24 (29.27%)	0.783
Toxic exposure [*n* (%)]	4 (7.41%)	7 (8.54%)	1.000
MSA subtype			0.686
MSA-C [*n* (%)]	38 (70.37%)	55 (67.07%)	
MSA-P [*n* (%)]	16 (29.63%)	27 (32.93%)	
Possible/probable (*n*)	24/30	26/56	0.132
Orthostatic symptoms [*n* (%)]	27 (50.00%)	55 (67.07%)	**0.046**
RBD	38 (70.37%)	64 (78.05%)	0.312
Urge incontinence	33 (61.11%)	50 (60.98%)	0.987
Constipation [*n* (%)]	37 (68.52%)	58 (70.73%)	0.783
UMSARS total score	39.87 ± 15.24	40.41 ± 13.96	0.725
UMSARS-I	20.98 ± 7.28	22.30 ± 6.39	0.240
UMSARS-II	19.06 ± 8.72	18.11 ± 8.17	0.653
UMSARS-IV	2.46 ± 1.28	2.43 ± 1.19	0.956
NMSS	32.24 ± 13.26	37.55 ± 10.27	**0.005**
MOCA	22.54 ± 4.62	23.40 ± 4.44	0.271
HCBS [*n* (%)]	34 (62.96%)	48 (58.54%)	0.606
Moderate-to-severe WMH [*n* (%)]	4 (7.41%)	9 (10.98%)	0.489

HCBS, hot cross bun sign; MoCA, Montreal cognitive assessment; MSA, multiple system atrophy; NMSS, non-motor symptoms scale; OH, orthostatic hypotension; RBD, REM sleep behavior disorder; UMSARS, unified MSA rating scale; WMH, white matter hyperintensity. Values are *n* (%) or mean ± SD. *p* values with statistical significance are shown in bold.

The multivariable binary logistic regression model (Table 4) included sex, age at onset, MSA subtype, UMSARS-I score, and NMSS score. Male (OR = 2.57, 95% CI = 1.21–5.44) and higher NMSS scores (OR = 1.06, 95% CI = 1.02–1.11) were independently associated with severe OH.

**Table 4:** Factors independently associated with severe OH among patients with OH in multivariable binary logistic regression analysis.

Variables	OR	95% CI	*p*
Male	2.57	1.21–5.44	**0.014**
Age of onset	0.98	0.93–1.03	0.450
MSA-C	1.06	0.46–2.44	0.888
UMSARS-I	0.98	0.91–1.05	0.617
NMSS	1.06	1.02–1.11	**0.009**

MSA, multiple system atrophy; NMSS, non-motor symptoms scale; UMSARS, unified MSA rating scale. *p* values with statistical significance are shown in bold.

In sensitivity analyses, the NMSS Domain 1 subscore was not significantly associated with severe OH (*p* > 0.05), whereas a modified NMSS total score excluding Domain 1 items remained independently associated with severe OH (*p* < 0.05), consistent with the primary analysis (Supplementary Table S2).

In subgroup analyses, male sex and NMSS score showed directionally consistent associations with severe OH across the MSA-C and MSA-P subgroups, though most estimates were not statistically significant (Figures 1A,B).

**Figure 1:**
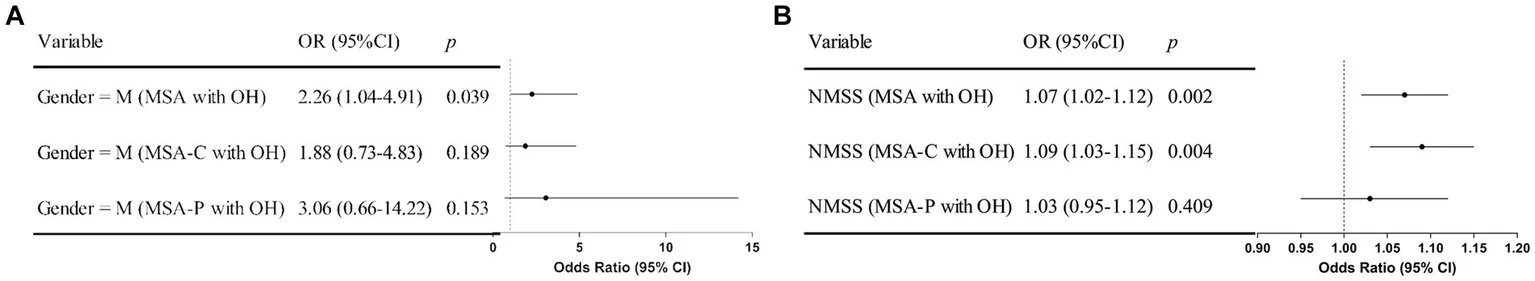
**(A)** Forest plot of the association between male sex and severe OH in the overall OH-positive cohort, MSA-C subgroup, and MSA-P subgroup. M, male; MSA, multiple system atrophy; OH, orthostatic hypotension. **(B)** Forest plot of the association between NMSS score and severe OH in the overall OH-positive cohort, MSA-C subgroup, and MSA-P subgroup. MSA, multiple system atrophy; NMSS, non-motor symptoms scale; OH, orthostatic hypotension.

## 4 Discussion

This cross-sectional study investigated factors associated with the presence and severity of OH in MSA. OH was present in 56.20% of the study population. In multivariable analyses, MSA-C subtype, probable MSA diagnostic certainty, and higher UMSARS-I scores were independently associated with the presence of OH. Among patients with OH, male sex and higher NMSS scores were independently associated with severe OH. The associations of male sex and NMSS score with severe OH were directionally similar in the overall OH-positive cohort and in the MSA-C and MSA-P subgroups; however, the subgroup-specific estimates should be interpreted cautiously because they did not reach statistical significance.

In our study, 56.20% of patients with MSA had OH. This finding is consistent with the study by Jiang et al. (12), which reported an OH prevalence of 51.58% among 444 MSA patients. Traon et al. (10) noted that 54% of patients had OH within 3 min of standing, this proportion increased to 72% when standing blood pressure was assessed beyond 10 min. These findings are also broadly consistent with the approximately 57% OH prevalence reported in a European multicenter cohort (18).

We found no significant difference in the prevalence of OH between male and female patients. However, male sex was more common among patients with severe OH than among those with mild OH, and was independently associated with severe OH in the multivariable analysis. This finding is consistent with that of Leys et al. (19), who reported an association between male sex and OH severity during longitudinal follow-up. Sex-related differences in vascular and autonomic responses may partly contribute to this association. For example, vascular responses to sympathetic stimulation may differ between men and women with aging (20). In addition, the use of alpha-adrenergic antagonists for male urinary tract symptoms, including benign prostatic hyperplasia, may aggravate orthostatic blood pressure reduction (12). However, medication exposure was not analyzed in the present study; therefore, this explanation remains speculative. In the stratified analyses, the point estimates were greater than 1 in both subgroups and were directionally consistent with the estimate in the overall OH-positive cohort, but the association between male sex and severe OH did not reach statistical significance in either the MSA-C or MSA-P subgroup. The lack of statistical significance may reflect limited subgroup sample sizes. These findings require confirmation in larger cohorts.

Only a limited number of studies have compared OH between the MSA-C and MSA-P subtypes. Wenning et al. (21) in a study of 16 patients with MSA-C and 17 patients with MSA-P, reported a higher prevalence of OH in MSA-C. Consistent with this observation, OH was more prevalent in MSA-C than in MSA-P in our study, and MSA-C subtype was independently associated with the presence of OH. In contrast, MSA subtype was not independently associated with severe OH among patients who already had OH. This finding is consistent with the study by Traon et al. (10), which reported no association between OH severity and MSA subtype, although it differs from the findings of Jiang et al. (12). The association between MSA-C subtype and the presence of OH may reflect differences in the distribution or extent of neurodegeneration between clinical subtypes. However, the present cross-sectional design does not permit conclusions regarding underlying mechanisms. The absence of an independent association between subtype and severe OH suggests that, once OH is present, factors other than clinical subtype may be more strongly associated with its severity. However, the predominance of MSA-C in our cohort reflects regional demographic characteristics and may limit the generalizability of our findings to populations with a higher MSA-P proportion.

Probable MSA was independently associated with the presence of OH. This finding should be interpreted in the context of the MSA diagnostic criteria, because objective autonomic failure, including OH, contributes to diagnostic certainty. Because OH itself contributes to the diagnostic certainty of probable MSA, the association between probable MSA and OH may be partly circular. Therefore, the observed association may partly reflect overlap between the diagnostic classification and the outcome definition. However, in a sensitivity analysis excluding diagnostic certainty, MSA-C subtype and UMSARS-I score remained independently associated with OH, indicating that the main findings were not caused solely by this overlap.

Urge incontinence and higher NMSS scores were associated with the presence of OH in the univariable analysis; however, these associations were not retained in the multivariable analysis. Thus, in this study, neither urge incontinence nor NMSS score was independently associated with the presence of OH. The lack of an independent association between urge incontinence and OH may be consistent with partially distinct underlying neural mechanisms. Sakakibara et al. (22) reported that neurogenic bladder dysfunction in MSA was not associated with OH. Although both manifestations reflect autonomic dysfunction, OH primarily reflects impairment of adrenergic pathways involved in cardiovascular regulation, whereas bladder dysfunction may involve the pons, sacral preganglionic neurons, and Onuf’s nucleus (23). However, the present cross-sectional findings cannot establish distinct pathophysiological mechanisms for OH and bladder dysfunction.

NMSS scores were higher in patients with OH than in those without OH and were also higher in patients with severe OH than in those with mild OH. Although NMSS score was not independently associated with the presence of OH, a higher NMSS score was independently associated with severe OH among patients with OH. In the stratified analyses, this association was statistically significant in the MSA-C subgroup. In the MSA-P subgroup, the association was directionally consistent but did not reach statistical significance, possibly because of the smaller sample size and limited statistical precision. The association between higher NMSS scores and severe OH may have several explanations. From a neuropathological perspective, OH may be related to degeneration of medullary sympathetic regulatory centers, whereas the autonomic symptoms assessed by the NMSS may reflect involvement of the pontomedullary autonomic network (24, 25). More extensive involvement of these regions may therefore be associated with both a greater non-motor symptom burden and more severe OH. In addition, MSA-C may involve the pons and medulla and may be accompanied by more prominent autonomic manifestations.

A seemingly unexpected finding was that the NMSS Domain 1 subscore was not significantly associated with severe OH, whereas the modified NMSS total score excluding Domain 1 remained independently associated. This result is consistent with previous evidence. A prospective study of patients with OH showed a poor relationship between the magnitude of the orthostatic BP fall, the upright orthostatic BP, and symptoms, and most patients were asymptomatic or only mildly symptomatic despite substantial blood pressure drops (26). Similarly, population-based data in older adults showed that two-thirds of individuals with OH did not report typical dizziness, and postural dizziness was not associated with the degree of orthostatic blood pressure fall. Wieling et al. showed that orthostatic symptoms correlate with the lowest blood pressure while standing, rather than with the magnitude of the blood pressure decrease (8). Another study found that patients with chronic orthostatic hypotension may be remarkably tolerant of low orthostatic blood pressure, developing no or few symptoms (27). Hypotensive unawareness is particularly common in synucleinopathies such as MSA, and preserved cerebral autoregulation may further reduce symptom perception despite marked blood pressure changes (27, 28). Because NMSS Domain 1 primarily captures patient-reported cardiovascular symptoms, it may not reliably reflect the severity of OH as defined by sphygmomanometric thresholds. In contrast, the modified NMSS score excluding Domain 1 reflects a broader non-motor symptom burden that may better parallel the overall extent of autonomic dysfunction and thus remains independently associated with severe OH. Future studies evaluating individual NMSS domains, or using measures with less symptom overlap, are warranted.

We did not observe statistically significant associations between OH and RBD, constipation, or cognitive impairment. This may reflect heterogeneous underlying mechanisms, limited statistical power, or characteristics of the present sample.

In the present study, OH status and severity were not independently associated with motor function or overall disability. Although patients with OH had higher UMSARS-I scores than those without OH, UMSARS-I includes urinary and orthostatic symptom items that may overlap with manifestations of OH (10), This overlap may partly explain the higher UMSARS-I scores observed in the OH group and is consistent with previous findings (12).

We found no independent associations of HCBS or moderate-to-severe WMH with either the presence or severity of OH. Anatomically, HCBS is considered to reflect degeneration of pontine transverse fibers, with pathological changes centered in the basis pontis (29). In contrast, WMH are predominantly located in the periventricular region and centrum semiovale, whereas autonomic structures relevant to OH include the ventrolateral medulla (30). Although the pons and medulla are both brainstem structures, they contain relatively distinct nuclei and neural pathways. Therefore, pontine structural changes do not necessarily indicate impairment of medullary sympathetic regulatory centers. In addition, WMH do not directly reflect involvement of autonomic pathways in the brainstem and spinal cord.

The lack of association may also reflect different pathological processes. WMH commonly reflect ischemic demyelination related to cerebral small-vessel disease, whereas OH in MSA is considered to result primarily from neurodegeneration associated with alpha-synuclein accumulation (31). Furthermore, some WMH in this cohort may have been attributable to age-related or vascular factors rather than MSA-related pathology, potentially attenuating any association with OH.

This study has several limitations. First, its cross-sectional design precludes inferences regarding temporality or causality. Second, the relatively small sample size, particularly in subgroup analyses, may have limited statistical power and the precision of the estimates. Third, the study was conducted in a single cohort, which may limit the generalizability of the findings. Multicenter studies with larger cohorts are needed to confirm these associations. Fourth, post-void residual volume was not assessed, which may have limited the comprehensive evaluation of lower urinary tract autonomic dysfunction. Fifth, we did not perform continuous beat-to-beat hemodynamic monitoring, dynamic trends of BP changes within the 3-min period were not formally analyzed. Finally, the onset and duration of supine hypertension and OH were not collected, which limited our ability to examine their temporal relationship and clinical progression.

## 5 Conclusion

In this cross-sectional cohort, factors associated with the presence of OH and severe OH were different. Neither HCBS nor moderate-to-severe WMH was significantly associated with the presence of OH.

## Data Availability

The raw data supporting the conclusions of this article will be made available by the authors, without undue reservation.

## References

[1] Jellinger KA. Multiple system atrophy: an oligodendroglioneural synucleinopathy1. J Alzheimer's Dis. (2018) 62:1141–79. doi: 10.3233/JAD-170397 PMC5870010 28984582

[2] Fanciulli A Wenning GK. Multiple-system atrophy. N Engl J Med. (2015) 372:249–63. doi: 10.1056/NEJMra1311488, 25587949

[3] Tada M Onodera O Tada M Ozawa T Piao YS Kakita A . Early development of autonomic dysfunction may predict poor prognosis in patients with multiple system atrophy. Arch Neurol. (2007) 64:256–60. doi: 10.1001/archneur.64.2.256, 17296842

[4] Glasmacher SA Leigh PN Saha RA. Predictors of survival in progressive supranuclear palsy and multiple system atrophy: a systematic review and meta-analysis. J Neurol Neurosurg Psychiatry. (2017) 88:402–11. doi: 10.1136/jnnp-2016-314956, 28250027

[5] Jain S Goldstein DS. Cardiovascular dysautonomia in Parkinson disease: from pathophysiology to pathogenesis. Neurobiol Dis. (2012) 46:572–80. doi: 10.1016/j.nbd.2011.10.025, 22094370 PMC3299874

[6] Colosimo C Morgante L Antonini A Barone P Avarello TP Bottacchi E . Non-motor symptoms in atypical and secondary parkinsonism: the PRIAMO study. J Neurol. (2010) 257:5–14. doi: 10.1007/s00415-009-5255-7, 19669613

[7] Ha AD Brown CH York MK Jankovic J. The prevalence of symptomatic orthostatic hypotension in patients with Parkinson's disease and atypical parkinsonism. Parkinsonism Relat Disord. (2011) 17:625–8. doi: 10.1016/j.parkreldis.2011.05.020, 21689962

[8] Wieling W Kaufmann H Claydon VE van Wijnen VK Harms MPM Juraschek SP . Diagnosis and treatment of orthostatic hypotension. Lancet Neurol. (2022) 21:735–46. doi: 10.1016/S1474-4422(22)00169-7, 35841911 PMC10024337

[9] Coon EA Ahlskog JE. My treatment approach to multiple system atrophy. Mayo Clin Proc. (2021) 96:708–19. doi: 10.1016/j.mayocp.2020.10.005, 33673922

[10] Pavy-Le Traon A Piedvache A Perez-Lloret S Calandra-Buonaura G Cochen-De Cock V Colosimo C . New insights into orthostatic hypotension in multiple system atrophy: a European multicentre cohort study. J Neurol Neurosurg Psychiatry. (2016) 87:554–61. doi: 10.1136/jnnp-2014-309999, 25977316

[11] Grimaldi S Boucekine M Witjas T Fluchere F Azulay JP Guedj E . Early atypical signs and insula hypometabolism predict survival in multiple system atrophy. J Neurol Neurosurg Psychiatry. (2021) 92:881–9. doi: 10.1136/jnnp-2020-324823, 33893230

[12] Jiang Q Zhang L Lin J Wei Q Li C Hou Y . Orthostatic hypotension in multiple system atrophy: related factors and disease prognosis. J Parkinsons Dis. (2023) 13:1313–20. doi: 10.3233/JPD-230095, 38143372 PMC10741317

[13] Gilman S Wenning GK Low PA Brooks DJ Mathias CJ Trojanowski JQ . Second consensus statement on the diagnosis of multiple system atrophy. Neurology. (2008) 71:670–6. doi: 10.1212/01.wnl.0000324625.00404.15, 18725592 PMC2676993

[14] Wenning GK Tison F Seppi K Sampaio C Diem A Yekhlef F . Development and validation of the unified multiple system atrophy rating scale (UMSARS). Mov Disord. (2004) 19:1391–402. doi: 10.1002/mds.20255, 15452868

[15] Horimoto Y Aiba I Yasuda T Ohkawa Y Katayama T Yokokawa Y . Longitudinal MRI study of multiple system atrophy – when do the findings appear, and what is the course? J Neurol. (2002) 249:847–54. doi: 10.1007/s00415-002-0734-0, 12140668

[16] Pradeep A Raghavan S Przybelski SA Preboske GM Schwarz CG Lowe VJ . Can white matter hyperintensities based Fazekas visual assessment scales inform about Alzheimer's disease pathology in the population? Alzheimer's Res Ther. (2024) 16:1. doi: 10.1186/s13195-024-01525-5, 38987827 PMC11234605

[17] Botz J Lohner V Schirmer MD. Spatial patterns of white matter hyperintensities: a systematic review. Front Aging Neurosci. (2023) 15:1165324. doi: 10.3389/fnagi.2023.1165324, 37251801 PMC10214839

[18] Wenning GK Geser F Krismer F Seppi K Duerr S Boesch S . The natural history of multiple system atrophy: a prospective European cohort study. Lancet Neurol. (2013) 12:264–74. doi: 10.1016/S1474-4422(12)70327-7, 23391524 PMC3581815

[19] Leys F Eschlböck S Campese N Mahlknecht P Peball M Goebel G . Sex-related differences in the clinical presentation of multiple system atrophy. Clin Auton Res. (2024) 34:253–68. doi: 10.1007/s10286-024-01028-1, 38630378 PMC11127878

[20] Baker SE Limberg JK Ranadive SM Joyner MJ. Neurovascular control of blood pressure is influenced by aging, sex, and sex hormones. Am J Physiol Regul Integr Comp Physiol. (2016) 311:R1271–5. doi: 10.1152/ajpregu.00288.2016, 27856414 PMC5256972

[21] Wenning GK Granata R Krismer F Dürr S Seppi K Poewe W . Orthostatic hypotension is differentially associated with the cerebellar versus the parkinsonian variant of multiple system atrophy: a comparative study. Cerebellum. (2012) 11:223–6. doi: 10.1007/s12311-011-0299-5, 21822547

[22] Sakakibara R Tateno F Yamamoto T Uchiyama T Yamanishi T. Urological dysfunction in synucleinopathies: epidemiology, pathophysiology and management. Clin Auton Res. (2018) 28:83–101. doi: 10.1007/s10286-017-0480-0, 29124503

[23] Benarroch EE. The clinical approach to autonomic failure in neurological disorders. Nat Rev Neurol. (2014) 10:396–407. doi: 10.1038/nrneurol.2014.88, 24866874

[24] Benarroch EE. Brainstem in multiple system atrophy: clinicopathological correlations. Cell Mol Neurobiol. (2003) 23:519–26. doi: 10.1023/a:1025067912199, 14514012 PMC11530157

[25] Luo Y Yang N Yang W Chen B Zhu S Wu Y . Autonomic dysfunction in multiple system atrophy: from pathophysiology to clinical manifestations. Ann Med. (2025) 57:1. doi: 10.1080/07853890.2025.2488111, 40719373 PMC11983539

[26] Freeman R Illigens BMW Lapusca R Campagnolo M Abuzinadah AR Bonyhay I . Symptom recognition is impaired in patients with orthostatic hypotension. Hypertension. (2020) 75:1325–32. doi: 10.1161/HYPERTENSIONAHA.119.13619, 32223377 PMC12818701

[27] Pavy-Le Traon A Hughson RL Thalamas C Galitsky M Fabre N Rascol O . Cerebral autoregulation is preserved in multiple system atrophy: a transcranial Doppler study. Mov Disord. (2006) 21:2122–6. doi: 10.1002/mds.21130, 17029266

[28] Lenka A Lamotte G Beach P. Asymptomatic orthostatic hypotension in synucleinopathies: to treat or not to treat? Clin Auton Res. (2024) 34:25–9. doi: 10.1007/s10286-023-01006-z, 38079008

[29] Prasad S Rossi M. The hot cross bun sign: a journey across etiologies. Mov Disord Clin Pract. (2022) 9:1018–20. doi: 10.1002/mdc3.13596, 36339309 PMC9631848

[30] Squair JW Berney M Castro Jimenez M Hankov N Demesmaeker R Amir S . Implanted system for orthostatic hypotension in multiple-system atrophy. N Engl J Med. (2022) 386:1339–44. doi: 10.1056/NEJMoa2112809, 35388667

[31] Udow SJ Robertson AD MacIntosh BJ Espay AJ Rowe JB Lang AE . ‘Under pressure’: is there a link between orthostatic hypotension and cognitive impairment in α-synucleinopathies? J Neurol Neurosurg Psychiatry. (2016) 87:1311–21. doi: 10.1136/jnnp-2016-314123, 27613160

