# Supplementary material for: Factors associated with orthostatic hypotension presence and severity in multiple system atrophy: a cross-sectional study
Source: Front Neurol. 2026 Sep 17;17:1950923. doi: 10.3389/fneur.2026.1950923 (PMC13626901; doi:10.3389/fneur.2026.1950923)
Supplement: Supplementary file 1 [file Table_1.DOCX]

Table S1. Sensitivity analysis for factors associated with OH presence in MSA patients after excluding diagnostic certainty.

| Analysis | OR (95% CI) | *p* |
| --- | --- | --- |
| MSA-C | 2.63 (1.46–4.72) | **0.001** |
| UMSARS-I | 1.11（1.04-1.18） | **0.002** |

Abbreviations: MSA, multiple system atrophy; UMSARS, unified MSA rating scale. p values with statistical significance are shown in bold.

Table S2. Sensitivity analyses for the association between NMSS and severe OH in OH-positive MSA patients.

| Analysis | NMSS variable | OR (95% CI) | *p* |
| --- | --- | --- | --- |
| Primary analysis | Total NMSS score | 1.07 (1.02–1.12) | **0.009** |
| Sensitivity analysis 1 | NMSS Domain 1 subscore | 1.20（0.97-1.48） | 0.087 |
| Sensitivity analysis 2 | NMSS total score excluding Domain 1 | 1.06（1.01-1.11） | **0.014** |

Abbreviations: MSA, multiple system atrophy; NMSS, non-motor symptoms scale. p values with statistical significance are shown in bold.
